# Lung disease assessment in primary ciliary dyskinesia: a comparison between chest high-field magnetic resonance imaging and high-resolution computed tomography findings

**DOI:** 10.1186/1824-7288-35-24

**Published:** 2009-08-06

**Authors:** Silvia Montella, Francesca Santamaria, Marco Salvatore, Marco Maglione, Paola Iacotucci, Maria Margherita De Santi, Carmine Mollica

**Affiliations:** 1 Department of Paediatrics, University of Naples Federico II, Naples, Italy; 2 Department of Diagnostic Imaging, University of Naples Federico II, Naples, Italy; 3 Department of Human Pathology and Oncology, University of Siena, Siena, Italy; 4 Biostructure and Bioimaging Institute, National Research Council, Naples, Italy; 5 IRCCS-SDN Foundation, Naples, Italy

## Abstract

**Background:**

Primary ciliary dyskinesia (PCD) is associated with pulmonary involvement that requires periodical assessment. Chest high-resolution computed tomography (HRCT) has become the method of choice to evaluate chronic lung disease, but entails exposure to ionizing radiation. Magnetic resonance imaging (MRI) has been proposed as a potential radiation-free technique in several chest disorders. Aim of our study is to evaluate whether high-field MRI is as effective as HRCT in identifying PCD pulmonary abnormalities. We also analyzed the relationships between the severity and extension of lung disease, and functional data.

**Methods:**

Thirteen PCD patients (8 children/5 adults; median age, 15.2 yrs) underwent chest HRCT and high-field 3T MRI, spirometry, and deep throat or sputum culture. Images were scored using a modified version of the Helbich system.

**Results:**

HRCT and MRI total scores were 12 (range, 6–20) and 12 (range, 5–17), respectively. Agreement between HRCT and MRI scores was good or excellent (r > 0.8). HRCT and MRI total scores were significantly related to forced vital capacity (r = -0.5, *p *= 0.05; and r = -0.7, *p *= 0.009, respectively) and forced expiratory volume at 1 second (r = -0.6, *p *= 0.03; and r = -0.7, *p *= 0.009, respectively).

**Conclusion:**

Chest high-field 3T MRI appears to be as effective as HRCT in assessing the extent and severity of lung abnormalities in PCD. MRI scores might be used for longitudinal assessment and be an outcome surrogate in future studies.

## Background

Primary ciliary dyskinesia (PCD; MIM #244400) is a rare (1:15–30 000 live births) and usually autosomal recessive disease associated with *situs viscerum inversus *(Kartagener syndrome) in nearly half the cases [1]. Impaired mucociliary clearance due to defective motility of cilia is the hallmark of the condition [2]. Early clinical events are represented by continuous rhinorrhoea from the first days of life, respiratory distress or neonatal pneumonia with no obvious predisposing cause, and chronic or recurrent lower and upper airway infections, e.g. otitis media and purulent rhinosinusitis [3]. Three lower airway diseases are associated with PCD: pneumonia, bronchiectasis, and asthma [3].

Computed tomography (CT) of the chest, particularly high-resolution CT (HRCT), has become the method of choice to evaluate chronic lung disease at any age [4,5]. Nevertheless, CT entails exposure to ionizing radiation, thereby increasing the risk of cancer in exposed individuals [6,7]. Magnetic resonance imaging (MRI) of the chest has been proposed as a potential radiation-free technique in several chest disorders [8-13]. However, its application in lung disease has long been limited by technical problems, namely a low signal-to-noise ratio because of the low proton density of the lung, and artifacts due to cardiac and breathing motion or to air/soft tissue transition [14]. Nevertheless, the results of chest MRI in assessment of lung disease were found to be comparable with those of conventional chest X-ray and CT, mainly in patients with cystic fibrosis (CF) [15-17]. Lung disease in PCD is similar to lung disease in CF, although changes can be milder in PCD [18]. Recent studies showed that CT scoring systems adequately describe the extent and severity of PCD lung changes [18-20]. Patients with PCD may develop chronic lower airways symptoms and/or signs at any time [21]. Therefore, a sensitive radiation-free imaging tool is highly desirable for the longitudinal assessment of structural lung damage.

To our knowledge, no study has compared chest CT to MRI in PCD. The primary aim of this pilot study was to assess whether MRI is as effective as CT in identifying pulmonary abnormalities in patients with PCD. Our secondary aim was to investigate the relationships between the severity and extension of lung disease, identified with HRCT and MRI, and pulmonary function tests (PFTs).

## Methods

### Patients

Thirteen subjects (8 children/5 adults; median age, 15.2 yrs; range: 10.4–29.3 yrs) with PCD followed at the Department of Paediatrics, University of Naples Federico II, Naples, Italy, were prospectively enrolled in the study. PCD was suspected on the basis of clinical features and/or *situs viscerum inversus *[3]. The clinical characteristics of the study population are summarized in Table 1. Diagnosis was confirmed by light microscopy (LM) and by electron microscopy (EM) analysis of cilia ultrastructure on nasal brushing at a median age of 7.3 yrs (range, 0.1–17.1 yrs). In all cases LM revealed dysmotility or immotility [see Additional file 1]. All patients were undergoing the following treatment: daily airway clearance therapy constituted by nebulized saline prior to chest physiotherapy; physical exercise; and aggressive treatment of upper and lower airway infections by antibiotics.

**Table 1:** Clinical characteristics of the study population

	**All** **(n = 13)**	**Children** **(n = 8)**	**Adults** **(n = 5)**
**Clinical data**			
**Male/Female**	9/4	6/2	3/2
***Situs viscerum inversus *(%)**	61	75	40
**Age at study entry (yrs)**	15.2 (10.4–29.3)	13.1 (10.4–17.7)	23.8 (20.9–29.3)
**Age at onset of respiratory symptoms (yrs)**	0.1 (0.1–4)	0.1 (0.1–4)	0.1 (0.1–1)
**Presenting respiratory symptoms**			
**Chronic cough (%)**	46	50	40
**Pneumonia (%)**	31	12.5	60
**Persistent wheezing (%)**	8	12.5	0
**Neonatal respiratory distress (%)**	15	25	0
**Age at PCD diagnosis (yrs)**	7.3 (0.1–17.1)	7 (0.3–15.5)	10.1 (0.1–17.1)
**Exhaled nitric oxide (ppb)**	4.3 (1.5–8.2)	5 (2.6–8.2)	3.9 (1.5–6.9)
**Nasal nitric oxide (ppb)**	15.8 (2.6–34.3)	12 (4.4–34.3)	16.9 (2.6–30.1)
**Atopy (%)**	23	25	20
**Lobectomy or segmentectomy (%)**	31	12	60

Values in parentheses are ranges.

Patients underwent chest HRCT for clinical reasons, namely persistence of chronic cough and/or focal abnormality at chest X-ray unresponsive to medical treatment, and/or discrepancy between lung function or clinical status and chest X-ray. In all patients chest MRI and HRCT were performed on the same day.

The ethics review board of the Medical School, University of Naples Federico II, Naples, Italy, approved the study, and informed, written consent was obtained from the parent/legal guardian of each child and from adult patients.

### HRCT scanning

The HRCT scan was performed with a 4-slice CT scanner (Aquilion, Toshiba, Japan) and a bodyweight adapted protocol (adults: 120 kV, 140 mAs; children over 45 kg: 120 kV, 65 mAs; children over 35 kg: 120 kV, 45 mAs; children below 35 kg: 120 kV, 30 mAs), with 1 × 4 mm collimation, 10 mm gap, 0.5 sec rotation time, automatic exposure control, multiple inspiratory breath holds of 3 sec each, with the patient in a supine position. The field of view of each sequence was patient-adapted. Images were reconstructed using a high-resolution algorithm. The total time for acquisition of the images was about 5 minutes, including positioning of the patient. Contrast medium was not administered. A lung window setting (+1500/-500 Hounsfield unit) was used for image analysis.

### MR scanning

MRI was performed with a 3T MR scanner (Magnetom Trio, Siemens Erlangen, Germany). We used a dedicated 12-element integrated matrix coil system, covering the whole thorax, for signal reception. It consisted of one anterior and one posterior flexible phased-array coil, each containing a set of six receiver elements. We applied the following sequences: 1) a half-Fourier single-shot turbo spin-echo (HASTE) sequence; 2) true-Fast Imaging with Steady Precession (true-FISP or TRUFI); and 3) volume-interpolated breath-hold examination (VIBE) sequence before and after (25, 60, and 180 sec) injection of contrast medium (0.1 mmol/kg gadopentetate dimeglumine). The field of view of each sequence was patient-adapted. HASTE and true-FISP/TRUFI sequences were performed using an electrocardiograph-gating to reduce cardiac motion artefacts, and respiratory-gating by a navigator signal that monitored the diaphragm position. Sequence parameters were: 1) HASTE: repetition time/echo time/flip angle, infinite/92 ms/150°; parallel acquisition factor, 2; slice thickness, 5 mm; distance factor, 20%; transversal (matrix, 380 × 256) and coronal (matrix, 400 × 320) orientation; acquisition time, approximately 90 sec; 2) True-FISP/TRUFI: repetition time/echo time/flip angle, 364.8–477.8 ms/1.2–4.3 ms/41–52°; parallel acquisition factor, 2; slice thickness, 5 mm; distance factor, 20%; transversal, coronal, and sagittal orientation (matrix, 380–480 × 320); acquisition time, 14–22 sec; 3) VIBE: repetition time/echo time/flip angle, 3.3 ms/1.2 ms/11.5°; parallel acquisition factor, 2; slice thickness, 3 mm; distance factor, 20%; transversal orientation (matrix, 350 × 256); acquisition time, 19 sec approximately.

No patient required sedation. Door-to-door time was approximately 15 min. All HRCT and MRI studies were of diagnostic quality and were well tolerated.

### Image evaluation

All identifying information was removed from the scans. To compare chest MRI and HRCT results, we scored HRCT and MR scans using a modified version of the scoring system developed by Helbich *et al*. for CF [22]. The original Helbich score includes severity and extent of bronchiectasis, severity of peribronchial wall thickening, extent of mucous plugging, generation of bronchial divisions involved by bronchiectasis or plugging, extent of sacculations or abscesses, severity of bullae, severity of emphysema, severity of collapse or consolidation, and severity of mosaic perfusion [22]. We excluded the severity of mosaic perfusion from imaging evaluation because it cannot be assessed by morphological MRI [16]. Therefore, the maximum total score was 25 points and not 27 (Table 2). We evaluated HRCT and MRI using the modified Helbich CT score as follows: 1) for the categories "severity of bronchiectasis" and "severity of peribronchial wall thickening", we recorded the most prevalent degree of severity; 2) it was not possible to assess peribronchial wall thickening in the presence of mucous plugging; 3) hyperintensity on HASTE images had to be present for an MRI diagnosis of mucous plugging; 4) if mucous plugging was seen within the periphery of a lung segment, bronchiectasis was scored also in that segment; 5) sacculations/abscesses were defined as circular structures with a minimum diameter of 1.5 cm that were air-filled or showed an air-fluid level; 6) a size of 2 cm was required for a diagnosis of collapse/consolidation; 7) emphysema was defined as an area of decreased signal (compared with the surrounding lung parenchyma) due to a reduction of vessel and parenchymal density; and 8) in case of lobectomy or segmentectomy, the maximum scores for "severity of bronchiectasis" and "severity of collapse/consolidation" were arbitrarily assigned to the missing lobe/segments. The assessment of "extent of bronchiectasis" took into account the number of missing segments.

**Table 2:** Modified Helbich scoring system for HRCT and MRI

	**Score**			
	
**Category**	**0**	**1**	**2**	**3**
Severity of bronchiectasis	Absent	Mild (lumen slightly greater than diameter of adjacent blood vessel)	Moderate (lumen 2 to 3 times the diameter of the vessel)	Severe (lumen > 3 times the diameter of the vessel)
Severity of peribronchial wall thickening	Absent	Mild (wall thickness equal to diameter of adjacent vessel)	Moderate (wall thickness greater than and up to twice the diameter of adjacent vessel)	Severe (wall thickness more than twice the diameter of adjacent vessel)
Extent of bronchiectasis	Absent	1–5^#^	6–9^#^	> 9^#^
Extent of mucous plugging	Absent	1–5^#^	6–9^#^	> 9^#^
Extent of sacculations or abscesses	Absent	1–5^#^	6–9^#^	> 9^#^
Generation of bronchial divisions involved (bronchiectasis or plugging)	Absent	Up to the 4^th ^generation	Up to the 5^th ^generation	Up to the 6^th ^generation and distal
Severity of bullae	Absent	Unilateral (not > 4)	Bilateral (not > 4)	> 4
Severity of emphysema	Absent	1–5^#^	> 5^#^	Not applicable
Severity of collapse or consolidation	Absent	Subsegmental	Segmental or lobar	Not applicable

^# ^Numbers of bronchopulmonary segments.

Six lobes were examined; the lingula was scored separately. In patients with *situs viscerum inversus*, the right lung was the lung in which the middle lobar bronchus and the corresponding middle lobe were identified at scans. The images were evaluated in consensus by two experienced observers (one radiologist with an 8-years experience and one paediatric pulmonologist with a 15-years experience in HRCT and MRI evaluation). Raters were blinded to the patients' history and to any clinical data that could have biased their interpretation of the images. Disagreement between the two observers occurred in only one case, and then the debated abnormality was scored by the most trained rater. MRI and HRCT scans were presented to the raters in a random, independent order. HRCT scans were scored 8 weeks after the MR images, so that the HRCT findings would not influence the raters' judgments of the MRI findings. MRI scores resulted from the combined evaluation of the three image sets (HASTE, True-FISP/TRUFI, and VIBE), because the information provided by each set is complementary to that deriving from the others.

### Lung function and microbiological evaluation

Forced vital capacity (FVC) and forced expiratory volume at 1 second (FEV_1_) were measured, on the same day as chest imaging, with spirometry according to ATS criteria [23]. A FEV_1 _> 85% predicted was considered normal. Deep throat or sputum cultures were also obtained in all patients at the time of HRCT and MRI evaluation.

### Statistical analysis

Results are expressed as median and range values. Spearman's rank correlation coefficient (rho) assessed correlations among the variables and agreement between HRCT and MRI scores. A coefficient of > 0.8 represents good agreement. A two-sided *p *≤ 0.05 was significant. Data were analyzed with SPSS-PC, release 13.0, SPSS Inc. (Chicago, IL).

## Results

Bronchiectasis, peribronchial wall thickening, mucous plugging and collapse/consolidation were the most frequent lung changes at HRCT and at MRI in the entire study population, and in children and adults (Table 3). MRI failed to detect bullae in two patients, but identified mucous plugging more frequently than HRCT. There were no other differences in the prevalences of lung abnormalities identified by the two techniques.

**Table 3:** Prevalence of abnormalities at HRCT and MRI in the study population

	**HRCT**			**MRI**		
	
	**All** **(n = 13)**	**Children** **(n = 8)**	**Adults** **(n = 5)**	**All** **(n = 13)**	**Children** **(n = 8)**	**Adults** **(n = 5)**
**Bronchiectasis (%)**	92	87	100	92	87	100
**Peribronchial wall thickening (%)**	100	100	100	100	100	100
**Mucous plugging (%)**	92	87	100	100	100	100
**Sacculations or abscesses (%)**	8	0	20	8	0	20
**Bullae (%)**	15	12	20	0	0	0
**Emphysema (%)**	15	12	20	15	12	20
**Collapse or consolidation (%)**	92	87	100	92	87	100

**Figure 1:**
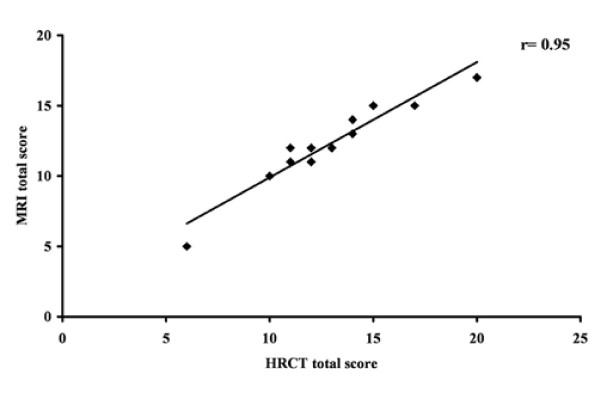
**Agreement between MRI and HRCT total scores in the whole population**. Twelve points are shown because of the overlap of one value.

Median HRCT and MRI scores are summarized in Table 4. Since only one or two patients had sacculations/abscesses, bullae or emphysema, agreement between HRCT and MRI scores was not calculated for these categories. Moreover, agreement for generation of bronchial divisions involved by bronchiectasis or plugging was not computable because of the constant value assigned to this category at HRCT and at MRI in all the subjects. However, the same score for generation of bronchial divisions involved by the above mentioned changes was attributed in each patient to both HRCT and MRI scans. For all the other categories, agreement between the two techniques was good or excellent. Figure 1 shows the excellent correlation (r = 0.95) between HRCT and MRI total scores.

**Table 4:** Median HRCT and MRI scores of the whole study population and their agreement

	**HRCT**	**MRI**	**r**
**Severity of bronchiectasis**	2 (0–3)	2 (0–3)	0.87
**Severity of peribronchial wall thickening**	2 (1–2)	2 (0–2)	1
**Extent of bronchiectasis**	2 (0–3)	2 (0–3)	0.97
**Extent of mucous plugging**	2 (0–3)	2 (1–3)	0.94
**Extent of sacculations or abscesses**	0 (0–1)	0 (0–1)	NA
**Generation of bronchial divisions involved** **(bronchiectasis or plugging)**	3*	3*	NA
**Severity of bullae**	0 (0–3)	0*	NA
**Severity of emphysema**	0 (0–1)	0 (0–1)	NA
**Severity of collapse or consolidation**	2 (0–2)	2 (0–2)	1

**Total score**	12 (6–20)	12 (5–17)	0.95

NA: not applicable (see text).

Values in parentheses are ranges.

* No range values are given because all patients had the same score. For generation of bronchial divisions involved by bronchiectasis or plugging, the same score was attributed in each patient to both HRCT and MRI scans.

Median FVC and FEV_1 _were 96% predicted (range, 57–121) and 85% predicted (range, 47–108), respectively. Children had higher values than adults for both FVC (96% predicted [range, 82–120] *versus *80% predicted [range, 57–121], respectively) and FEV_1 _(89% predicted [range, 72–108] *versus *60% predicted [range, 47–103], respectively). Total HRCT and MRI scores were significantly related to FVC (r = -0.5, *p *= 0.05; and r = -0.7, *p *= 0.009, respectively) and FEV_1 _(r = -0.6, *p *= 0.03; and r = -0.7, *p *= 0.009, respectively). Fifty-four percent of patients had a normal FEV_1_.

The most common pathogen at deep throat or sputum culture was *Haemophilus influenzae *in both adults and children (60% and 50%, respectively). *Pseudomonas aeruginosa *was isolated only in one children and in one adult, whereas *Staphylococcus aureus *was found in one boy. In three cases no pathogens were cultured.

**Figure 2:**
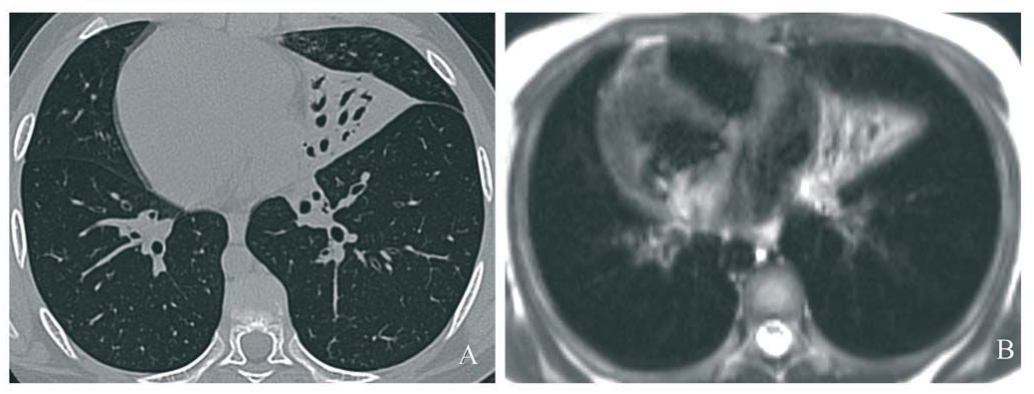
**Transversal CT image (A) and transversal MRI HASTE (B) sequence of a 10.4-year-old boy with Kartagener syndrome**. The scans demonstrate an area of consolidation in the middle lobe.

Figure 2 shows an area of consolidation at HRCT (panel A) and MRI (panel B) scans of a boy with PCD and *situs viscerum inversus *(Kartagener syndrome).

## Discussion

In this study, we compared the efficacy of chest HRCT and high-field MRI in the assessment of the severity and extent of lung abnormalities in children and young adults with PCD. The agreement between the two techniques was good or excellent for most of the abnormalities considered. Bronchiectasis, peribronchial wall thickening, mucous plugging, and collapse/consolidation were the most frequent abnormalities. HRCT and MRI total scores were significantly related to PFTs.

At present, the management of patients with PCD is based on clinical assessment and regular sputum cultures combined with pulmonary function evaluation [3]. Pulmonary function measurement is generally used to assess lung disease in PCD. It has been previously reported that lung function assessment, and particularly static lung volumes evaluation, may provide suggestive information about peripheral airway disease and its evolution over time [3,21,24-26]. However, PFTs are relatively insensitive markers of early disease and fail to detect regional structural changes because these tools reflect the function of the lung as a whole and gives no information about localized abnormalities. Chest CT provides a more accurate picture of the type, distribution and nature of lung changes than either PFTs or chest radiography [4,27]. In fact, we recently found that 40% of PCD patients had a normal FEV_1 _despite CT evidence of significant structural lung damage [18]. Our MRI results reported herein confirm this finding in that pulmonary abnormalities were easily recognized also in patients with normal lung function.

Chest CT has the substantial advantage of revealing early and/or localized structural changes [14,28]. Nevertheless, despite its potential benefits, CT is not regularly used in PCD, and there is no consensus as to when it is best obtained [3]. However, although the outcome of PCD is not universally unfavourable, complications of bronchiectasis and severe pulmonary impairment become more severe with age [21]. Early assessment of structural lung changes is then crucial in PCD, especially in candidates for lobectomy and/or lung transplantation [29]. CT has been criticized for its ionizing radiation burden and the possible consequences of cumulative doses, particularly when used for frequent follow-up examinations in patients with chronic lung diseases, in pregnancy, or in children [6,7]. Several surveys on the effects of repeated CT examinations have demonstrated that lifetime cancer risks are cumulative and not negligible, even though the consequences of low levels of exposure have not been clearly elucidated [30,31]. It has been hypothesized that, at low doses, there is a linear dose-response relationship between the exposure to ionizing radiation and the development of solid cancers in humans [32]. It is unlikely that there is a threshold below which cancers are not induced, but at low doses the number of radiation-induced cancers might be small. In addition to this, other health end-points have been linked to radiation exposure. In particular, statistically significant associations have been found with heart diseases, stroke, and diseases of the digestive, respiratory, and hematopoietic systems [32]. In this scenario, a sensitive technique that could assess PCD lung disease without ionizing radiation is highly desirable.

To determine the clinical value of chest MRI scanning and its potential as a surrogate outcome measure in PCD, we assessed the comparability of MR and HRCT images using a quantitative scoring system, i.e. a modified Helbich score [16]. Although the Helbich score was devised for the CF setting [22], we used it because there is no scoring system specific for PCD and because we found it to be easily adaptable to MRI. Here we show that in PCD patients the results of chest MRI coincide with chest CT findings. Indeed, we found an excellent agreement between HRCT and MRI for most of the categories of the scoring system, thereby supporting the concept that MRI might be an alternative, radiation-free method for pulmonary assessment in PCD as well as in CF [15-17]. This issue is particularly relevant in PCD because of the presumed long life expectancy of these patients.

Chest MRI has several advantages over CT. First, being radiation-free, it can be repeated (e.g., in case of artifacts), and is thus suitable for long-term disease monitoring. Second, MRI identifies various characteristics of lung tissue, allows a precise characterization of the lesion and assesses function, e.g. lung perfusion and/or ventilation, and respiratory mechanics [14]. Thus, structural information and functional data are obtained in a single examination, thereby reducing imaging costs and increasing the patient's compliance [33,34]. Finally, unlike CT, chest MRI easily distinguishes between mucous plugging and bronchial wall thickening even in the peripheral airways [15]. This finding is relevant because peripheral mucous plugging is a sensitive marker of early small airway disease in PCD [18]. None of these features is found (or is only partly found) in spirometry and other surrogate outcome measures, including CT.

On the other hand, the spatial resolution of MRI is lower than that of CT and slight morphological changes such as peripheral bronchiectasis without bronchial wall thickening are not consistently visualized by MRI [15]. However, this did not seem to affect our findings as shown by the comparable HRCT and MRI scores. Other potential drawbacks of MRI are limited access to the technology, long acquisition times and high costs. Long acquisition times represented a concern in our study, because our population included children who were unable to hold their breath for a long time, despite training. Therefore, we used respiratory-gating to overcome the lack of cooperation in children, and applied it also to adults for the sake of uniformity. We believe that the benefits of MRI outweigh the greater discomfort for the patient and the higher costs. Furthermore, technical progress will hopefully lead to lower costs, shorter examination times and higher image resolution.

To our knowledge, this is the first time that 3T MRI has been used for the quantitative analysis of pulmonary abnormalities in children and adults with PCD. Moreover, this study is the first to compare typical PCD findings at chest CT with MRI. It is also the first evaluation of chest high-field 3T morphological MRI in patients with lung disorders due to abnormal mucociliary clearance. Although a field strength of 1.5T is generally used in clinical practice, MR whole-body units operating at field strengths of 3T and beyond are increasingly being installed in research institutions and in clinical facilities [35-37]. The scanner we used has several advantages over 1.5T: 1) it has a high-speed and a high-strength gradient system; 2) it is equipped with multiple phased-array coils and receiver channels; and 3) it has acquisition acceleration techniques, such as parallel imaging. Parallel acquisition techniques improve image quality by shortening the echo times of single-shot sequences [38,39]. Despite the high-field used, shorter echo times result in decreased blurring artifacts and less signal decay caused by T2* effects. Another advantage of the system we used is that cardiac- and respiratory-gating reduce artifacts due to heart/great vessels/chest wall motion thereby overcoming the need for sedation even in poorly cooperating subjects. Thus, patient's discomfort was less with this system. However, our study is limited by the fact that CT scans were performed for clinical purposes, images were read in consensus, functional MRI data were not provided, infants were not included, and longitudinal data were not obtained. In addition to this, MRI failed to detect bullae in two of our patients. In both of them these lesions were small in size, and it has been previously reported that small pulmonary bullae might pose a problem to chest MRI [40]. However, the prevalence of bullae in our study population was very low, and other authors failed to detect them at HRCT in larger PCD populations [19,20]. Given the very low prevalence of bullae in PCD and the small size of the lesions found in our patients, the failure in the detection of bullae unlikely entails a significant clinical impact. Nevertheless, further studies on chest MRI in larger PCD populations are needed.

## Conclusion

Chest high-field 3T MRI appears to be as effective as HRCT in the assessment of extent and severity of PCD lung disease. Therefore, it might be considered a reliable radiation-free option to HRCT in PCD and be proposed for follow-up examinations. This non-ionizing radiation technique could prove to be a useful tool in monitoring lung disease, guiding clinical management and assessing the consequences of therapy in PCD as well as in other pulmonary disorders in children, adults and the elderly.

## Abbreviations

PCD: Primary ciliary dyskinesia; CT: Computed tomography; HRCT: High-resolution computed tomography; MRI: Magnetic resonance imaging; CF: Cystic fibrosis; PFT: Pulmonary function test; LM: Light microscopy; EM: Electron microscopy; HASTE: Half-Fourier single-shot turbo spin-echo; True-FISP/TRUFI: True-Fast Imaging with Steady Precession; VIBE: Volume-interpolated breath-hold examination; FVC: Forced vital capacity; FEV_1_: Forced expiratory volume at 1 second.

## Competing interests

All authors declare that they have not been funded and they disclose any involvement with organisation(s) with financial interest in the subject matter or materials discussed in the submitted manuscript. All authors declare that they have no potential conflict of interest, real or perceived.

## Authors' contributions

SM participated in the design of the study, performed the statistical analysis, and drafted the manuscript. FS conceived of the study, participated in its design and coordination, and scored HRCT and MR images. MS conceived of the study and participated in its design. MM and PI participated in the collection of data and helped to draft the manuscript. MMDS participated in the design of the study and performed the electron microscopy analysis of cilia ultrastructure. CM carried out the imaging studies, scored the HRCT and MR images, and helped to draft the manuscript. All authors read and approved the final manuscript.

## Supplementary Material

Additional file 1 **Cilia ultrastructure at EM and motion pattern at LM of the study population**. The data provided in the table derive from individual cilia ultrastructural analysis at EM and evaluation of ciliary motion pattern at LM. Click here for file
