# Supplementary material for: Lung disease assessment in primary ciliary dyskinesia: a comparison between chest high-field magnetic resonance imaging and high-resolution computed tomography findings
Source: Ital J Pediatr. 2009 Aug 6;35:24. doi: 10.1186/1824-7288-35-24 (PMC2737542; doi:10.1186/1824-7288-35-24)
Supplement: Additional file 1 — Cilia ultrastructure at EM and motion pattern at LM of the study population. The data provided in the table derive from individual cilia ultrastructural analysis at EM and evaluation of ciliary motion pattern at LM. [file 1824-7288-35-24-S1.doc]

**Additional file 1.** Cilia ultrastructure at EM and motion pattern at LM of the study population.

| Case no. | Ultrastructural defects at EM | | | | | LM ciliary motion pattern^§^ |
| --- | --- | --- | --- | --- | --- | --- |
|  | Dynein arm  abnormalities^†^ | Peripheral  microtubule  abnormalities^†^ | Central pair  abnormalities^†^ | Compound cilia^†^ | Basal bodies  abnormalities^‡^ |  |
| 1 | Absent inner arm (89%) | Outer doublets  disarranged (10%) | Absent central pair (15%)  Microtubule  translocation,  9 + 0 (20%) |  |  | Immotile |
| 2* | Absent inner arm (95%) |  |  | 20% |  | Immotile |
| 3 | Absent inner and outer arm (100%) |  |  | 10% |  | Immotile |
| 4* |  | Outer doublets  disarranged (5%) | Absent central pair (35%) | 8% |  | Motile (Metronome) |
| 5* | Absent inner arm (100%) |  |  |  |  | Immotile |
| 6 | Absent outer arm (100%) |  |  |  |  | Immotile |
| 7 | Partial inner and outer arm defect (100%) |  |  |  |  | Immotile |
| 8 | Absent outer arm (95%)  Short inner arm (60%) |  |  | 10% |  | Immotile |
| 9 | Absent inner and outer arm (100%) |  |  | 10% |  | Immotile |
| 10* | Absent inner and outer arm (95%) | Outer doublets disarranged (3%) | Microtubule  translocation,  9 + 0 (15%) | 5% |  | Immotile |
| 11* | Absent inner and outer arm (98%) |  | Microtubule  translocation,  9 + 0 (2%) |  |  | Immotile |
| 12 | Absent outer arm (95%)  Short inner arm (80%) | Outer doublets  disarranged (5%) |  |  |  | Immotile |
| 13 |  |  |  |  | 100% | Immotile |

*Cases with *situs solitus*.

^†^ % cilia counted.

^‡^ Expressed as percentage of ciliated cells counted.

^§^ Ciliary beat frequency was < 7 Hz in all cases (normal range in our laboratory: 9,7-18,8 Hz).
